# A Causation Analysis of Chinese Subway Construction Accidents Based on Fault Tree Analysis-Bayesian Network

**DOI:** 10.3389/fpsyg.2022.887073

**Published:** 2022-06-03

**Authors:** Zijun Qie, Huijiao Yan

**Affiliations:** Faculty of Humanities and Social Sciences, Dalian University of Technology, Dalian, China

**Keywords:** Chinese subway construction, construction safety, cause factors, fault tree analysis, Bayesian-network

## Abstract

Clarifying the causes of subway construction accidents has an important impact on reducing the probability of accidents and protecting workers’ lives and public property to a greater extent. A total of 138 investigation records of subway construction accidents from 2000 to 2020 were collected in this study. Based on a systemic analysis of 29 well-known accident causation models and the formative process of the subway construction accidents, we extracted the causative factors of subway construction accidents from the collected records. Furthermore, a causation analysis index system of subway accidents was proposed based on fault tree analysis (FTA), where we considered subway construction accidents as the top event and the five dimensions, i.e., human, equipment, environment, management, and safety culture, as first-level intermediate events. Moreover, 17 causative factors were considered to be related to the severity of subway construction accidents. It is found that human factors are prone to be critical to high-risk accidents. Finally, a Bayesian network (BN) was formed to explore the causative factors of high-risk subway construction accidents. Based on the combined application of FTA and BN, this study discusses the complex influence factors and their action routes to unsafe accidents in subway construction sites, and makes efforts to correspond safety decision basis for the management of China subway construction.

## Introduction

The subway, as a symbol of modern metropolis development, plays a critical role in reducing traffic congestion, improving urban structure, and developing regional economies. Since the implementation of the New Infrastructure Construction policy in 2020, China has accelerated the development of the rail transit industry, and subway construction plans have gradually expanded from large-sized cities to medium-sized cities. As of 31 December 2021, subways have been in operation in 40 cities in mainland China, covering a total distance of 7,253.73 km (China Association of Metros, 2022). Meanwhile, subway constructions are prone to safety accidents for their complex construction environment, strict technical requirements, and high safety risks. The threat to workers’ lives and public property cannot be ignored. Therefore, strengthening the management and control of causative factors for subway construction accidents has become an urgent research topic.

The desire for safety drives people to learn from past accidents and experiences. The investigation reports and related records of subway construction accidents are valuable and precious in identifying the causes of subway construction accidents and improving the management capacity of accident prevention. Therefore, this study tried to collect the subway construction accident cases in China on record from 2000 to 2020 as the sample data. Combined the practice cases with classic accident-causing models, a causation mechanism tree of subway construction accidents was constructed by using the fault tree analysis method (FTA), which is helpful to comprehensively identify the causative factors of subway construction accidents in China. Furthermore, a Bayesian network (BN) was applied to dynamic causation analysis and risk inference for high-risk subway construction accidents. We hope this study will be conducive to reducing the risks in subway construction projects and providing decision support for the safety management of infrastructure construction in China.

The remainder of this study is organized as follows: First, we discussed the literature review. Then, we described the data acquisition and analysis framework, and developed a causation analysis index system of subway construction accidents based on FTA. Furthermore, we analyzed the causation of high-risk subway construction accidents, where a BN model is presented. Finally, the conclusion and further study are presented.

## Literature Review

Understanding the accident-causing mechanism has always been considered as a prerequisite to prevent accidents, and it will benefit the improvement of construction management and technology. Therefore, a number of accident causation models have been developed in view of different fields.

**FIGURE 1:**
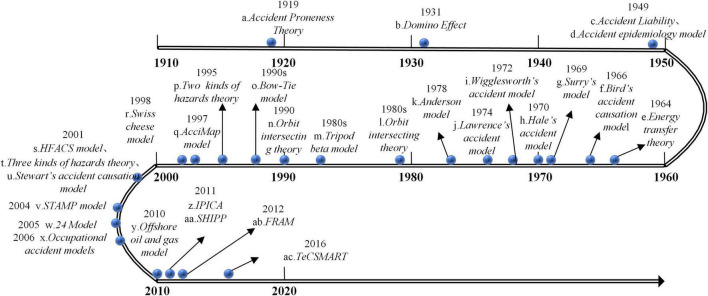
Accident causation model diagram.

Accident causation models, which are important theoretical bases and research methods in safety science, reflect a systematic analysis of the occurrence, development, and consequence of an accident (Fu et al., 2019). Figure 1 shows some emerging classic accident causation models over the past 100 years, which can be divided into four categories, namely, human error models, simple linear models, complex linear causation models, and systemic models (Katsakiori et al., 2009; Khanzode et al., 2012; Fan et al., 2014; Fu et al., 2019; Woolley et al., 2019; Wu et al., 2020). These accident causation models examine the causes of accidents from the initial worker factors, equipment factors to gradually breaking through the restrictions of the workplace, spreading to distant factors such as organization, safety atmosphere, and social environments (Khosravi et al., 2014).

In addition, the accident causation models have become more networked and multidimensional, showing the evolution process like “point-line-surface-space” (Huang and Wu, 2017). For existing accident causation models, they are proposed at certain historical periods, under specific circumstances and assumptions, hence different models come with various research emphases (Huang L. et al., 2020). However, among these models, the discussion on human errors (e.g., worker factors and management factors) is never absent. It means that human-related factors are always considered to be fundamental to accidents. For example, Hung et al. (2011) adopted a triangulation design consisting of observation, subjective quantitative, and subjective qualitative methods, concluding that safety problems and dangerous behaviors are affected by construction workers’ safety attitudes. Williams et al. (2018) studied accident causation classification and categorized it under five factors, namely, client-related, consultant-related, contractor-related, construction workers-related, and construction site-related. Clarke (2013) explored the impact of different styles of leadership on safety using meta-analytic path analysis.

The uncertainty associated with the underground construction environment (Seo and Choi, 2008) makes subway construction far more complex and generates much more serious accident consequences than general construction projects (Xing et al., 2019). Moreover, subway, as a critical infrastructure essential to social and economic development, has specific characteristics in its construction process compared to general construction projects, as well as in multiple stakeholders such as government, enterprises, and citizens. At present, accident studies in the field of public infrastructure construction like subway mainly focus on technical, geological, and environmental safety risk factors (Ding et al., 2012). Since accidents in complex environments are resulted from multiple causes combined (Chen et al., 2020), a comprehensive review of subway construction accident risk factors is warranted. Some studies explored the multiparty or multilevel causative factors in a major subway construction accident with a certain accident causation model. For instance, Niu et al. (2016) established a structural model of subway construction safety control based on the STAMP model, which is used to investigate the causal factors of the 2008 Hangzhou subway collapse. Besides, another group of studies investigated the causative factors through a large number of subway construction accident cases. Typically, Zhou et al. (2021) developed a network of SCSRN to integrate causations with various accidents on subway construction sites. He (2018) coded 57 subway construction accidents in China based on grounded theory, and extracted accident causal factors into four core genera, i.e., management, human, environmental condition, and physical factors.

In conclusion, there are already several classic accident causation models which can help a lot when clarifying the causes of subway construction accidents. At the same time, due to the specific characteristics of subway construction projects, it is worth noting that the adaptability and pertinence of these models for subway construction accident analysis should be further optimized. The study of influencing factors based on only subway accident data often leads to ignoring the gradual development process of accidents. Therefore, we intended to explore an integrated way, in which accident causation models can be adjusted by combining with actual details of the subway construction accidents, and the static analysis of the causative factors, as well as dynamic prediction, can be accomplished. For this purpose, we introduced a hybrid approach with FTA and BN. The literature review on accident causation models provides theoretical support for establishing the causation index system of subway construction accidents.

## Methodology

After a major accident, most countries will provide accident investigation reports to the public. Although the standards of accident report preparation differ from country to country, the report’s core components are generally similar. A typical accident investigation report not only provides a summary and recommendations on the consequences of the accident, but also documents the details of the safety event and the determining factors that may cause the accident. We planned to use the information extracted from the accident investigation report for data analysis. Therefore, the data collection and processing of subway construction incidents are discussed in the following sections.

### Data Acquisition

The scope of the data was the subway construction accidents occurred between 2001 and 2020. The provinces (autonomous regions and province-level municipalities) for the accident collection were only within mainland China, excluding Hong Kong, Macau, and Taiwan Province. The data were mainly collected from Emergency Management Bureau websites, Housing and Urban and Rural Construction Bureau websites at various levels, official media, security forums, etc. Finally, 138 investigation reports of subway construction accidents with relatively comprehensive information were acquired. There were eight non-production safety liability accidents. For the remaining 130 subway construction accident records, there were 109 low-risk accidents (ordinary accidents) and 21 high-risk accidents. The statistical items of cases collected are shown in Table 1.

**TABLE 1:** Statistics of accident cases.

Accident code	Subway line	Time	Accident consequences	Accident level	Detailed description of the cases (data source)
1	Shanghai	2001-8-20	Four people killed	Lager accident	http://www.riskmw.com/case/2010/07-23/mw22487.html
2	Line 1, Hangzhou	2008-11-15	Twenty one people were killed; 24 people injured; direct economic loss reached 49.61 million Yuan	Major accident	http://www.hangzhou.gov.cn/art/2017/7/9/art_1256343_8272707.html
3	Line 4, Suzhou	2016-8-4	One person killed; direct economic losses amounted to about 1.05 million Yuan	Ordinary accident	http://yjglj.suzhou.gov.cn/szsafety/sgdccl/201612/2b34b904f2ff48cc8110f93e5aaff377.shtml
…	…	…	…	…	…
138	Line 4, Shenzhen	2020-7-29	One person killed; direct economic loss reached 1.8 million Yuan	Ordinary accident	http://www.szlhq.gov.cn/zdlyxxgk/aqsc/dcbg/content/post_8176254.html

*Accident levels are classified according to the Regulations on the Reporting and Investigation of Workplace Accidents in China.*

### Analysis Framework

Fault tree analysis is a graphical interpretation method that can capture the causes of system failures or the probability of accidents. It uses logical symbols to link system failures and the factors that cause them and operate based on Boolean logic, which is considered to be an effective way of system security assessment (Lawrence and Gill, 2007). The fault tree analysis method was developed by the telephone laboratory of Bell Telegraph Company in 1962 and was originally applied in the fields of aerospace, military, nuclear energy, and navigation. Subsequently, as the method became more influential, it was introduced into cross-disciplinary areas (Wang et al., 2019; Liu et al., 2021).

Fault tree analysis takes the most undesired event of the system as the target of fault analysis and looks for all factors that directly cause this occurring fault event. The analysis goes down in sequence until those factors are found for which the probability distribution is known and no further exploration is required. A complete fault tree analysis method generally includes the following steps: establishment of fault trees, normalization of fault trees, qualitative analysis, quantitative analysis, etc. Researchers can operate several or all of these steps according to the study demands. “The causation analysis index system of subway construction accidents based on fault tree analysis” section uses the fault tree method, establishing a subway construction accident causation analysis index system.

The BN, also known as the Bayesian belief network, is composed of network nodes, directed edges, and conditional probability table (CPT), which is the product of the combination of probability theory and graph theory. It uses directed acyclic graphs (DAG) to qualitatively describe the dependencies among nodes and the conditional probability distribution or CPT of each node to quantitatively express the influence relationships among nodes, providing reliability in the problems with uncertainty and incompleteness through limited samples or missing data (Xie, 2015). The Bayesian network is based on the Bayesian inference formula. For the event “*L*”, assuming that the sum of all events affecting its occurrence is *X* = (*X*_1_, *X*_2_, *X*_3_, … *X*_*n*_), the Bayesian formula can be calculated as follows:


(1) $$P\left(X_{i}|L\right)=\frac{P\left(L|X_{i}\right)P\left(X_{i}\right)}{P\,\left(L\right)}=\frac{P\left(L|X_{i}\right)P\left(X_{i}\right)}{\sum_{j=1}^{n}\left(L|X_{j}\right)P\left(X_{j}\right)},i=1,2,3,\ldots ,n,n\in N^{+}$$

where *X*_*i*_is the causative factor in the set *X.P*(*X*_*i*_) is the prior distribution, generally obtained from expert experience or historical data statistics. *P*(*L*|*X*_*i*_) is the likehood function. *P*(*L*) is the probability of the occurrence of event *L*, which can be calculated by the full probability formula. *P*(*X*_*i*_|*L*) is the posterior distribution.

**FIGURE 2:**
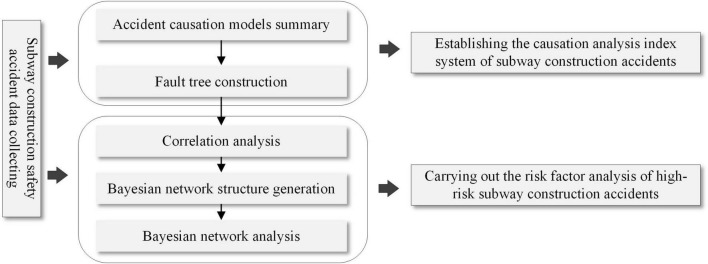
Framework for data analysis.

The above ones are the main technical methods to solve the problems in “The causation analysis index system of subway construction accidents based on fault tree analysis” and “Causation analysis of high-risk subway construction accidents based on Bayesian networks” sections, and our analysis process is shown in Figure 2. First, we collected the case data of subway construction accidents during recent 20 years. Then we developed a causation analysis index system of subway construction accidents based on fault tree analysis. In this process, we extracted the primary indicators from the classical accident causal models, which provide the theoretical support for the fault tree construction. Furthermore, correlation analysis was used to select the causative factors associated with high-risk subway construction accidents. Finally, a BN is established for the dynamic causation analysis and risk inference of high-risk subway construction accidents.

### The Causation Analysis Index System of Subway Construction Accidents Based on Fault Tree Analysis

According to the principle of fault tree construction, the “occurrence of subway construction accident” is regarded as the top event (T) of the fault tree which should be subdivided into basic risk-causing factors. We first summarized some classical accident causation models (shown in Figure 1). Based on these classic models, the causes of subway construction accidents were refined into five dimensions, namely, worker factors, equipment factors, environmental factors, organizational management factors, and safety culture factors (see Table 2), which constitute the first layer of intermediate events of the fault tree. Furthermore, the following subsections introduce the classification standards of secondary and tertiary indicators. Based on the historical subway construction accident reports, the primary indicators were progressively classified into 33 basic factors, so as to establish the causation analysis index system of subway construction accidents in China.

**TABLE 2:** The first-level intermediate events of fault tree analysis (FTA).

Accident causation model	Causation dimension
*a; b; c; f; g; h; i; j; l; n; q; r; s; u; v; w; x;* and *aa*	Worker factors
*b; f; h; l; u; w;* and *ac*	Equipment factors
*b; c; f; l; m; q; r; s; x; z;* and *ac*	Environmental factors
*e; f; h; j; l; n; q; r; s; u; v; w; x; z; aa;* and *ac*	Organizational management factors
*s; u; w;* and *x*	Safety culture factors

*The numbers “a-ac” in this table correspond to the numbers “a-ac” in Figure 1.*

#### Worker Factors

Worker factors refer to the direct operators, which are classified into unsafe characteristics and unsafe behaviors concerning the Human Factors Analysis Classification System (HFACS). The unsafe characteristics of workers include the poor psychological state and poor physical state. As for workers’ unsafe behaviors, we referred to the studies of Reason (1990); Man (2013), and Ma et al. (2014) to divide them into two categories, namely, unintentional and intentional. Unintentional unsafe behavior refers to an incorrect or improper response to external stimuli due to the actor’s perception bias, judgment error, and information transmission error, i.e., the actor’s next operation is often a natural instinctive reaction (Jiang, 2021), for example, working under a crane weight without realizing a crane weight in front of them, not knowing the safety hazards, or not wearing a helmet due to insufficient safety knowledge. Intentional unsafe behaviors refer to the behaviors with a clear purpose such as saving time and effort, or knowingly violating the rules (Chen et al., 2007). For example, during the construction process, though workers know that some behaviors do not meet the safety standards, they still take those fluke behaviors, including going to work after drinking, working when fatigued, and violating regulations after receiving complete safety education and training.

#### Equipment Factors

It can be observed from the accident cases that the equipment factors mainly refer to the failure of machinery equipment, consisting of the following two situations: the equipment running with disease due to negligence of daily inspection and maintenance, and the sudden failure of equipment due to equipment design defects or improper use and other reasons.

#### Environmental Factors

Subway construction is mostly carried out in underground spaces, which are narrow and easily affected by the surrounding geological environment, pipeline factors, ground traffic disturbance, and weather. The environmental factors that affect subway construction accidents can be divided into two categories, namely, organizational internal environmental factors and organizational external environmental factors. Among them, organizational internal environmental factors mainly refer to the problems in the operating environment of the workplace, such as cross-operation on the construction site, irregular placement of materials and equipment, low lighting, lack of monitoring equipment, and other safety hazards. Furthermore, the external environmental factors of the organization are divided into natural environmental factors, technical environmental factors, and policy environmental factors. Among them, natural environment factors mainly involve unfavorable geological and hydrological environments as well as meteorological factors. Surface subsidence and building damage accidents caused by poor geological and hydrological environments frequently occur in the process of subway construction in China. In addition, extreme weather, such as heavy rain, increases not only the difficulty of construction but also the probability of accidents by worsening the geological and hydrological conditions. Technical environmental factors mean the environmental conditions that are unfavorable for construction caused by human activities, which mainly refer to the ground vibration caused by human activities and the complex, unexplored or old underground pipelines that affect the construction. Policy environment factors refer to the subway accident occurred when related technology, standards, and norms have not been issued, thus making the subway construction in an environment without evidence.

#### Organizational Management Factors

Organizational management factors can directly or indirectly cause the system in a failure state, thereby increasing the probability of accidents (Xiao and Li, 2007). Therefore, it has become the consensus of most accident models to regard organizational management factors as the deep-seated causes of accidents. In essence, organizational management factors belong to human factors. Although they are partially identical, the mechanisms of organizational management errors are much more complex than those of individual mistakes. For the classification of organizational management factors, this study classifies them concerning the Plan Do Check Act (PDCA) method (Li and Huang, 2018), which divides management activities into four stages: Plan, Do, Check, and Act, and regards management activities as a continuous process that runs continuously in a cycle. Since the data collected in this study contain no factors related to “Act,” we divided the organizational and management factors of subway construction accidents into three categories, namely, Plan, Do, and Check, according to the research purpose.

When defining the Plan factors for subway construction accidents, we interpreted them as insufficient construction objectives, plans, methods, and specific measures formulated. Specifically, it is divided into three sub-dimensions, namely, insufficient foreseeability of safety accidents, planning and design deficiencies, and construction plan deficiencies. Among them, inadequate foreseeability of safety accidents refers to the situation where the subway construction unit misjudges the overall risk of construction due to weak safety awareness or a lack of relevant knowledge. Deficiencies in planning and design include defects in construction scheme design, confusion in the management of construction plans, and so on. The construction plan deficiency mainly includes the following three situations, i.e., the complete lack of the construction flow scheme, the partial lack of the construction scheme, and the complete construction scheme which is equal to the lack because of the vague expression.

The Do phase of the PDCA cycle is the stage of strict implementation of the formulated objectives and plans, and the implementation factors leading to the occurrence of subway construction safety accidents can be summarized into six sub-dimensions, namely, insufficient emergency response capabilities, deviation of construction plan execution, inadequate communication procedures, contract management deviation, subcontract management loopholes, and resource management disorder. Insufficient emergency response capabilities in subway construction accidents are mainly manifested by the absence of emergency plans and improper emergency handling at construction sites. Deviation of construction plan execution refers to the deviation or violation of construction plan in the process of subway construction, including the violation of technical regulations, violation of construction procedures, inadequate safety hazard inspection, and inappropriate implementation of technical delivery. Inadequate communication procedures mainly refer to the communication problems existing in the subordinate and subordinate departments and the departments at the same level in the process of subway construction. Contract management deviation refers to the situation that there are obvious errors in the process of contract formulation procedures and contents as well as obvious deviations in contract implementation during the subway construction process. Subcontracting management loopholes mainly refer to illegal subcontracting and escrow by subcontracting in the process of subcontracting. Resource management disorder includes two situations, i.e., human resource management disorder and material management disorder.

The Check factors leading to subway construction accidents mainly include three sub-dimensions, namely, inadequate construction monitoring, inadequate project supervision, and inadequate government supervision. Among them, inadequate construction monitoring refers to the following situations: inadequate supervision and management of the construction site by the construction unit, inadequate ivariables are treatnternal supervision and inspection of the project, and inadequate monitoring of the construction site. Moreover, inadequate project supervision refers to problems such as unqualified supervisors, absence of supervisors during inspections, and ineffective performance of supervisors in the process of third-party supervision. Inadequate government supervision mainly refers to the existence of poor communication and coordination between enterprises and government departments, imperfect relevant government rules and regulations, problems with enterprises in permitting construction procedures, the ineffective performance of relevant government departments, lax inspections, etc.

#### Safety Culture Factors

**FIGURE 3:**
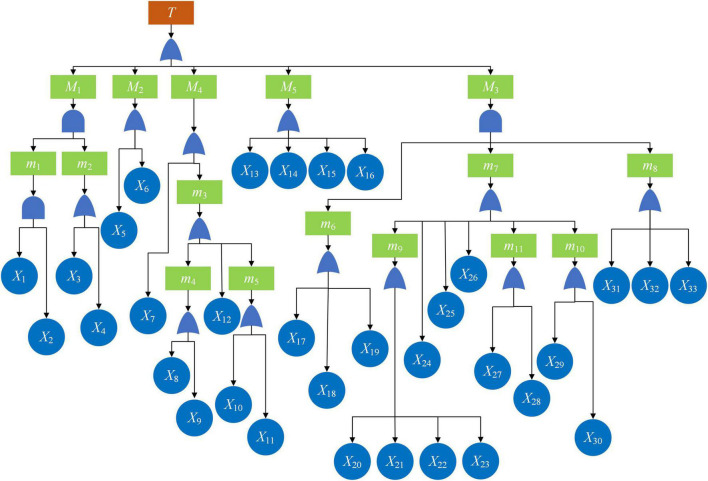
Subway construction accident causation mechanism tree.

“Safety culture” is the guiding ideology of safety operations at the organizational level (Fu et al., 2013), a term that has rapidly gained acceptance and has become popular among safety researchers worldwide since it was first used in the report of the International Nuclear Safety Advisory Group (INSAG) in 1986 (David and Zhang, 2021). Over the years, different scholars have formed different opinions on the connotation of safety culture. As safety culture is a subset of organizational culture; this study draws on Schein’s three-level model of organizational culture, i.e., organizational culture includes shallow worker artifacts, implicit values, and the deepest basic assumptions (Yan et al., 2015). We considered that the safety culture factors leading to subway construction accidents can be divided into three categories, namely, implementation level, management level, and system level. Specifically, according to the accident cases, they are summarized as non-execution of safety education and training, inadequate safety management systems, management’s lack of attention to safety production, and failure to implement safety regulations and systems. Among them, non-execution of safety education and training and the failure to implement safety regulations and systems refer to safety culture factors at the implementation level. Non-execution of safety education and training mainly means that safety training for workers is not carried out or perfunctory. Failure to implement safety regulations and systems refers to the failure of management personnel to implement relevant laws, norms, or regulations. Management’s lack of attention to safety production is a factor at the management level, including the risky construction ordered by managers in pursuit of economic benefits, the long-term absence of site duties, “Three Violations” activities, and so on. The inadequate safety management system is a factor at the system level, which mainly includes the insufficient rules and regulations and unclear management structure of the enterprise. Figure 3 shows the “subway construction accident causation mechanism tree,” which demonstrates the causative factor index system of subway construction accidents in China, and Table 3 provides a detailed supplement to Figure 3.

**TABLE 3:** Subway construction accident causation index explanation.

Index number	Causative factor	Index number	Causative factor
*M*_1_	Worker factors	*X*_10_	Unfavorable geological and hydrological environments
*M*_2_	Equipment factors	*X*_11_	Meteorological factors
*M*_3_	Organizational management factors	*X*_12_	Policy environmental factor
*M*_4_	Environmental factors	*X*_13_	Management’s lack of attention to safety production
*M*_5_	Safety culture factors	*X*_14_	Inadequate safety management system
*m*_1_	Workers’ unsafe characteristics	*X*_15_	Failure to implement safety regulations and systems
*m*_2_	Workers’ unsafe behavior	*X*_16_	Non-execution of safety education and training
*m*_3_	Organizational external environmental factors	*X*_17_	Insufficient foreseeability of safety accidents
*m*_4_	Technical environmental factors	*X*_18_	Planning and design deficiencies
*m*_5_	Natural environmental factors	*X*_19_	Construction plan deficiencies
*m*_6_	Plan factors	*X*_20_	Violation of technical regulations
*m*_7_	Do factors	*X*_21_	Violation of construction procedures
*m*_8_	Check factors	*X*_22_	Inadequate safety hazard inspection
*m*_9_	Deviation of construction plan execution	*X*_23_	Inappropriate implementation of technical delivery
*m*_10_	Insufficient emergency response capabilities	*X*_24_	Inadequate communication procedures
*m*_11_	Resource management disorder	*X*_25_	Contract management deviation
*X*_1_	Workers’ poor psychological state	*X*_26_	Subcontract management loopholes
*X*_2_	Workers’ poor physical state	*X*_27_	Human resource management disorder
*X*_3_	Intentional unsafe behavior	*X*_28_	Material management disorder
*X*_4_	Unintentional unsafe behavior	*X*_29_	Absence of emergency plans
*X*_5_	Equipment running with disease	*X*_30_	Improper emergency handling at construction sites
*X*_6_	Sudden failure of equipment	*X*_31_	Inadequate construction monitoring
*X*_7_	Organizational internal environmental factors(problems in the operating environment of the workplace)	*X*_32_	Inadequate project supervision
*X*_8_	Ground vibration caused by human activities	*X*_33_	Inadequate government supervision
*X*_9_	Underground pipeline factors		

## Causation Analysis of High-Risk Subway Construction Accidents Based on Bayesian Networks

According to the Regulations on the Reporting and Investigation of Workplace Accidents (The Central People’s Government of the People’s Republic of China, 2007), accidents that result in less than three deaths, fewer than 10 serious injuries, or less than 10 million Yuan in direct economic losses are defined as ordinary accidents. They account for the largest proportion of workplace accidents, and we defined them as low-risk accidents. As mentioned above, the accident consequences are generally measured by casualties and property losses. When the number of fatalities reaches three or more, it means that the accident reaches the level of a major accident. It is prone to significant negative social impacts. Although such accidents occur infrequently, they will cause great physical losses and hidden damage to the reputation of the project team and local government. Accidents of this level are defined as high-risk accidents in this study, e.g., the Qingdao subway collapse on 27 May 2019, which formed a superimposed effect of public opinion with the self-report of subcontractor, and caused strong concerns and doubts from public opinion about the project parties and Qingdao municipal government (Zhang and Shao, 2019). Therefore, much attention should be given to the high-risk subway construction accidents, particularly the management and control of related risk factors associated with subway construction accidents at this level.

**FIGURE 4:**
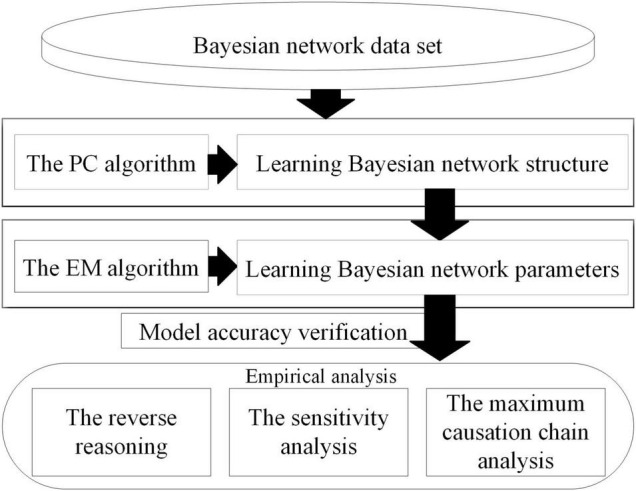
The Bayesian network build process.

In this section, the risk factors related to the severity of subway construction accidents are extracted from the subway construction accident causation index system through correlation analysis. Then the dataset is matrixed and a Bayesian network is constructed to analyze the causes of high-risk subway construction accidents. The Bayesian network is obtained in GeNIe3.0, including structure learning, parameter learning, model accuracy verification, and empirical analysis. The BN build process is shown in Figure 4.

### Correlation Analysis of the Causative Factors of High-Risk Subway Construction Accidents

As many as 33 basic causative factors of subway construction accidents were identified using fault tree analysis, which makes it difficult to guarantee the accuracy of BN constructed using these factors. To simplify the model structure and improve the efficiency of the BN structure learning, before learning the Bayesian network structure, we first conducted a correlation analysis between each causal variable and the variable “Severity of Subway Construction Accidents” (Li et al., 2014; Zhang and Sheng, 2019), so as to delete variables that are not strongly correlated with the study variable. For the correlation analysis, the severity of subway construction accidents was used as the dependent variable, while the other 33 causative factors were used as the independent variables. These variables are treated as dummy variables in SPSS. Herein, the occurrence of high-risk subway construction accidents is defined as “1”, and the low-risk subway construction accidents are defined as “0”; for the 33 risk factor variables, the two-state values of “occurred” and “not occurred” are presented with “1” and “0”, respectively. SPSS.26 was used to carry out the correlation analysis between each variable and low-risk subway construction accidents. As shown in Table 4, there are 17 causative factors related to the severity of subway construction accidents.

**TABLE 4:** Correlation analysis.

Causal variable	Correlation	Causal variable	Correlation	Causal variable	Correlation
*X*_1_	–0.039	*X*_12_	0.350**	*X*_23_	–0.052
*X*_2_	0.072	*X*_13_	0.249**	*X*_24_	0.017
*X*_3_	–0.148	*X*_14_	0.157	*X*_25_	0.322*
*X*_4_	–0.132	*X*_15_	–0.012	*X*_26_	0.063
*X*_5_	0.217*	*X*_16_	0.083	*X*_27_	0.346**
*X*_6_	–0.048	*X*_17_	0.375**	*X*_28_	0.154
*X*_7_	0.024	*X*_18_	0.180*	*X*_29_	0.365**
*X*_8_	0.238*	*X*_19_	0.004	*X*_30_	0.450**
*X*_9_	–0.037	*X*_20_	0.322**	*X*_31_	0.217*
*X*_10_	0.334**	*X*_21_	0.205*	*X*_32_	0.393**
*X*_11_	0.322**	*X*_22_	0.093	*X*_33_	0.299**

*p < 0.05; **p < 0.01.

### Establishment of the Causative Factor Matrix

Before constructing the Bayesian network model of high-risk subway construction accidents, we need to preprocess the data obtained combining with the results of correlation analysis. As shown in Eq. (2), *x_i_*indicates the causative factor *i*, where *i* ∈ {1,2,3,…,*n*}, *n* is the total number of causative factors related to the severity of subway construction accidents, and *n* ∈ *N*^+^; c_*k*_ represents the accident case *k*, where*k* ∈ {1,2,3,…,m}, *m* is the number construction accidents, and m ∈ *N*^+^. Then we can build the causative factor matrix *U*_*mn*_ as follows:


(2) $$U_{\mathrm{mn}}=\left[\begin{array}{ccc} U_{\mathrm{c1x1}} & \cdots & U_{\mathrm{c1xn}}\\ \vdots & \ddots & \vdots \\ U_{\mathrm{cmx1}} & \cdots & U_{\mathrm{cmxn}} \end{array}\right]$$

$$W\,h\,e\,r\,e\,U_{c\,k\,x\,i}=\left\{ \begin{array}{c} y\,e\,s,x_{i}\,o\,c\,c\,u\,r\,s\,i\,n\,a\,c\,c\,i\,d\,e\,n\,t\,c_{k}\\ n\,o,x_{i}\,d\,i\,d\,n\,o\,t\,o\,c\,c\,u\,r\,i\,n\,a\,c\,c\,i\,d\,e\,n\,t\,c_{k} \end{array}\right.,$$

$$\forall x_{i},i\in 1,2,3,\ldots ,n,n\in N^{+},$$

(3) $$\forall c_{k},k\in 1,2,3,\ldots ,m,m\in N^{+}$$

### Bayesian Network Empirical Analysis

The establishment of the BN structure plays an important role in the validity of the model, which is the critical part of Bayesian network analysis. At present, there are generally three ways to establish a Bayesian network structure: learning algorithms based on sample data, expert knowledge in the case of missing data or clear causality, or using the two methods together.

**FIGURE 5:**
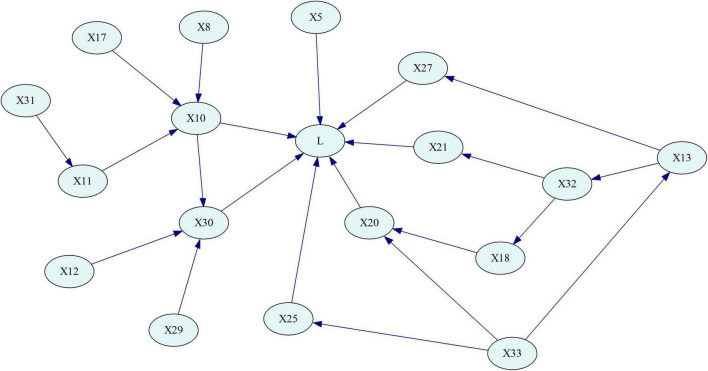
Bayesian network structure learning results.

Concerning the three methods mentioned, obtaining the Bayesian network structure through a dataset-based learning algorithm can reduce the influence of the researchers’ subjective factors to a large extent. However, with a limited data access, the effect of the structure learned only by data is not perfect, so it is necessary to add expert knowledge to adjust and revise the model. The PC algorithm is a classic learning algorithm based on sample data, which can separate the conditional independence relationship from the network structure search, and is closer to Bayesian semantic features in principle (Li, 2014). Therefore, this study chose PC algorithm to learn the BN structure, and adjusted the model through correlation analysis. Finally, the BN is obtained as shown in Figure 5, where “*L*” represents the severity of the subway construction accident.

**FIGURE 6:**
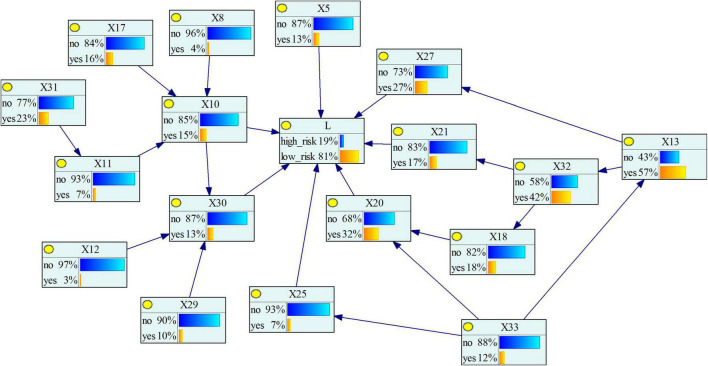
Bayesian network parameter learning results.

We can learn the parameters of each variable based on the acquired sample data to obtain the CPT of each network node in the model. As the parameters of BN, CPT can quantitatively analyze the correlation between nodes. In general, Bayesian parameter learning methods include EM algorithm, maximum likelihood method, Bayesian estimation method, etc. (Zhang et al., 2012). Our data were collected from real accident investigation reports, in which there may exist incomplete records and a limited sample size of the reports. In this case, the EM algorithm can make up for the insufficiency of the report and accurately reflect the probability value between nodes. Meanwhile, the convergence speed of EM is faster (Huang W. C. et al., 2020). Hence, we chose the EM algorithm for parameter learning and obtain the Bayesian network parameter learning results (Figure 6).

The prediction accuracy of the model was calculated by “K-fold cross-validation” of the GeNie software, whose prediction accuracy is 0.8846150. It means that our model has high prediction accuracy and can be used for causation analysis and model inference.

#### The Reverse Reasoning

**FIGURE 7:**
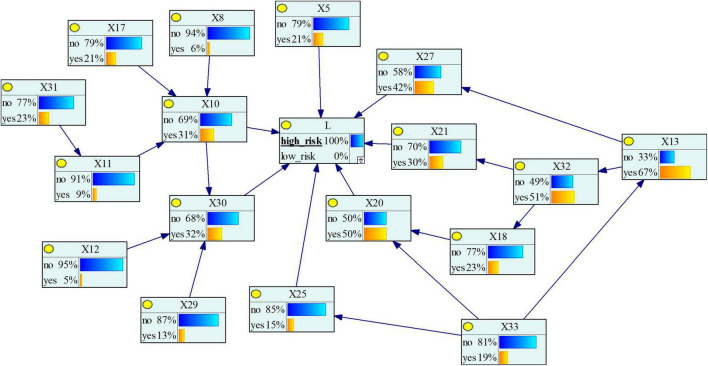
Bayesian network reverse reasoning results.

**FIGURE 8:**
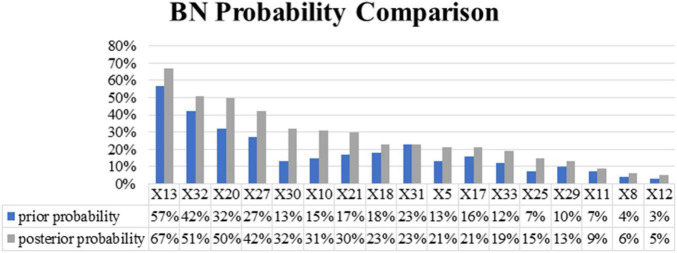
Bayesian network probability comparison.

Reverse reasoning is the most common conditional probability reasoning method for Bayesian network models, that is, by pre-setting the risk level of the severity of subway construction accidents, the probability of each causative factor under this level is reasoned, so as to diagnose the most likely cause of the risk level accident. After reverse reasoning, we can calculate the posterior probability of each factor, and then determine the importance of the causative factors to the target node. Factors with a large posterior probability have a greater impact on the resulting events and are the goals we should focus on controlling and improving. The posterior probability of each causative factor is shown in Figure 7, and its comparison with the change of the prior probability is shown in Figure 8. As can be seen from the figure, the posterior probability of the four causative factors *X*_13_, *X*_32_, *X*_20_, and *X*_27_ is higher when a high-risk subway construction accident occurs. In other words, when high-risk subway construction accidents occur, the four risk-causing factors, i.e., management’s lack of attention to safety production, inadequate project supervision, violation of technical regulations, human resource management disorder are more likely to occur. Among them, the consistent causative factor “*X*_13_” has the highest prior probability and posterior probability, and is the most likely key risk factor for accidents. This situation shows a series of behaviors such as project leaders not being at the construction site, ordering “Three Violations” (i.e., surveying, designing, and constructing at the same time) of construction. For example, an accident investigation report indicates that “the construction project team directed workers to take risks despite the existence of obvious accident hazards in the pit”, which is one of the direct causes of the accident.

#### The Sensitivity Analysis

**FIGURE 9:**
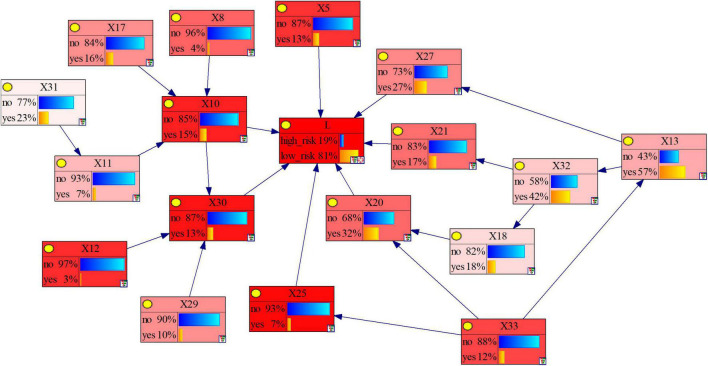
Bayesian network sensitivity analysis.

We used node sensitivity to describe how slight changes in one node’s parameters affect the output probability of other nodes in the BN. Identifying key causative factors only by prior or posterior probabilities may lead to inaccurate results, while sensitivity analysis can help for validation (Zarei et al., 2017). In a BN, the high-sensitivity causative factor nodes have a more significant impact on the result node, and by controlling the high-sensitivity causative nodes, the accident risk can be reduced with less cost. Figure 9 shows the results of Bayesian network sensitivity analysis overall, and the risk factors in the model are highly sensitive. Among them, the six causative factor nodes, i.e., *X*_5_, *X*_10_, *X*_12_, *X*_25_, *X*_30_, and *X*_33_ are in dark red. In other words, it is easier to reduce the risk by controlling the six risk factors of causative factors. The results show that in addition to improving management measures such as prevention and supervision, strengthening monitoring and early warning of the subway construction environment are also important ways to avoid the occurrence of high-risk subway construction accidents.

#### The Maximum Causation Chain Analysis

By setting “*L*” in the Bayesian network to “high risk” (Figure 7), and then searching for the node with the highest posterior probability of each child node in turn from this node, a complete accident propagation chain can be obtained. Among the child nodes of *L*, the posterior probability of *X*_20_ is the largest (50%). By reverse search in turn, the maximum causation chain of high-risk accidents can be obtained, that is, “*X*_33_→*X*_13_→*X*_32_→*X*_18_→*X*_20_→*L*”. It can be seen that to avoid the occurrence of large and above subway construction accidents, the key is to improve management and government supervision. For example, in the collapse of the Hangzhou Subway, there were problems with the supervision of Hangzhou Metro Group Co., Ltd. by the Hangzhou Municipal Government, which failed to observe that Hangzhou Subway Line 1 was not organized and implemented in strict accordance with the capital construction procedures (*X*_33_). The project leader ordered illegal construction and risky operations in order to catch up with the construction schedule (*X*_13_), while the supervision unit that should ensure the quality and safety of the project was absent (*X*_32_), resulting in serious over-excavation of the foundation pit and serious defects in the support system (*X*_20_). Ultimately, the superposition and coupling of multiple factors caused this collapse accident that shocked the world.

## Discussion and Conclusion

Due to the significant threat to public safety, social stability, and workers’ rights, the subway construction accident has aroused widespread concern and questioning in society (The Central People’s Government of the People’s Republic of China, 2013). Therefore, it is urgent to identify the key causative factors of subway construction accidents and to control them based on a thorough understanding of the accident mechanism.

A hybrid model based on FTA-BN was constructed in this study, and the following conclusions were obtained through theoretical and empirical analyses:

(1) A total of 33 essential indicators affecting subway construction accidents were identified through FTA and can be classified into five dimensions, namely, worker factors, equipment factors, environmental factors, organizational management factors, and safety culture factors.
(2) The correlation between the causative factors and the severity of subway construction accidents was established. The correlation analysis shows that there are 17 risk factors related to the severity of subway construction accidents. Among them, broader human errors, such as the lack of monitoring by government department staff, risky decisions by leaders, or even the lack of communication between two enterprises of the project team, play an important role. Therefore, in addition to focusing on preventing unsafe behaviors of frontline workers, subway construction practitioners should further investigate the root causes of accidents in order to seek interventions.
(3) A PC-EM-BN model was developed for the causal derivation and prediction of high-risk subway construction accidents. Through reverse reasoning, it is inferred that the key causative factor most likely to cause accidents is the factor “Management’s lack of attention to safety production.” Sensitivity analysis shows that the control of the six risk factors “*X*_5_, *X*_10_, *X*_30_, *X*_12_, *X*_25_, and *X*_33_” often achieves maximum results with less effort. Besides, maximum causation chain analysis shows that government supervision will play a significant role in reducing the probability of high-risk accidents, which is also confirmed by some studies (Xu, 2009; Yang et al., 2011; Bu and Li, 2021). Hence, government-related departments need to make more efforts in improving the design of the safety production supervision system, strengthening the communication and inspection with enterprises, etc.

In conclusion, subway construction, as a kind of typical public infrastructure construction, often involves more complex social environments and a wider range of stakeholders. It means that the accident causation is more complex. However, the studies available are still insufficient to explore the causal mechanisms of such accidents. Our study aims to propose a basic framework for subway construction practitioners through the combination of accident causation models and FTA, and expands the application scope of the BN to quantitatively predict the probability distribution of subway construction engineering safety performance. This is a meaningful attempt to combine accident causation theories and accident data for analysis, which also has significant practical implications for the security sector to formulate risk control policies. However, several limitations still exist. First, this is an attempt to analyze the causation of subway construction accidents in the Chinese context, and there is no accessible comparative analysis of related studies to validate the results. We hope that our findings will motivate researchers to further focus on construction safety management in related fields. Besides, the knowledge of practical and theoretical experts familiar with subway construction has been used relatively insufficiently in this study, which is planned to supplement in the further study. Moreover, how to optimize the combination of FTA and the BN is still an issue worthy of exploration and attention when complex factors intertwined. In future study, we will explore other methods to optimize the calculation of CPT during the conversion of FTA to the BN, to further explore the mechanism of risk factors in subway construction accidents.

## Data Availability Statement

The original contributions presented in this study are included in the article/supplementary material, further inquiries can be directed to the corresponding author.

## Author Contributions

ZQ designed the study, structure of the manuscript, and revised the original draft. HY wrote the original draft and performed the analysis. Both authors contributed to this article and agreed to the submitted version.

## Conflict of Interest

The authors declare that the research was conducted in the absence of any commercial or financial relationships that could be construed as a potential conflict of interest.

## Publisher’s Note

All claims expressed in this article are solely those of the authors and do not necessarily represent those of their affiliated organizations, or those of the publisher, the editors and the reviewers. Any product that may be evaluated in this article, or claim that may be made by its manufacturer, is not guaranteed or endorsed by the publisher.
